# Gastric Xanthoma Associated with Gastric Cancer Development: An Updated Review

**DOI:** 10.1155/2020/3578927

**Published:** 2020-02-21

**Authors:** Faycal Awaleh Moumin, Abdimajid Ahmed Mohamed, Abdirahman Ahmed Osman, Jianting Cai

**Affiliations:** Department of Gastroenterology, The Second Affiliated Hospital, School of Medicine, Zhejiang University, Hangzhou, Zhejiang Province 310009, China

## Abstract

Gastric xanthelasma (GX) is a rare tumor-like lesion customarily found as an incidental finding due to its asymptomatic appearance. Grossly, it is a well-marked yellow-white plaque created in the lamina propria by microscopic clusters of foamy macrophages. Xanthelasma is rarely correlated with gastric hyperplastic polyps; gastric xanthomas are rare benign lesions that appear to be associated with inflammation of the gastric mucosa. Etiopathogenesis is also unclear, but it has been suggested to be involved in chronic gastritis, infection with *Helicobacter pylori* (*H. pylori*), diabetes mellitus, and hyperlipidemia. The gastric xanthoma prevalence ranges from 0.23% to 7%. Orth first described the condition in 1887. It has been found that xanthelasmas are associated with chronic gastritis, gastrointestinal anastomosis, intestinal metaplasia, and *H. pylori* infection. These lesions predispose patients to gastric cancer conditions. Xanthoma (GX) was reported to be a predictive marker for early gastric cancer. However, the effectiveness of these scores and xanthoma (GX) as predictive markers for early gastric cancer detected after *H. pylori* eradication remains unknown.

## 1. Introduction

Xanthomas, also known as “xanthelasmas,” are plaque-like red lesions defined by the presence of histiocytic containing lipids. Incidentally, they are discovered during the upper endoscopy of the GI. In the upper GI tract, the most prevalent xanthomas place is in the abdomen (76%) [1]. Orth defined the condition first in 1887 [2]. Gastric xanthomas incidence varies from 0.23% to 7% [1, 3]. Although the prevalence of gastric xanthoma (GX) is a variety from different studies has been made, most xanthomas are rare lesions, often most common in the antral and pyloric areas. Colonic xanthelasmas were seldom found. They are less found in the esophagus, representing 12% [1]. Gastric xanthoma (GX) is typically asymptomatic and incidentally discovered for a multitude of indications on esophagogastroduodenoscopies (EGDs). However, symptoms such as abdominal pain or dyspepsia and abdominal distention, which prompt endoscopic assessment, are unlikely to be caused by rare gastric xanthoma (GX) lesions. As is often seen in patients with early stomach cancer in the gastric mucosa, xanthelasma was thought to be a predicative causative agent or gastric adenocarcinoma pre-tumorigenesis change [4, 5]. With pathological modifications such as chronic gastritis, intestinal metaplasia, atrophic gastritis, gastric ulcer, and alterations induced by bile reflux or partial gastrectomy, lipid islands are most frequently discovered in the stomach. Phagocytosis of *H. pylori* bacteria may induce the transformation of macrophages into foamy cells that penetrate the lamina. Therefore, this recent study shows that gastric xanthoma has a relationship with gastric cancer development. With pathological modifications such as chronic gastritis, intestinal metaplasia, atrophic gastritis, gastric ulcer, and alterations induced by bile reflux or partial gastrectomy, lipid islands are most frequently discovered in the stomach. Orth defined condition first in 1887 [6]. Gastric xanthoma, a localized nonneoplastic accumulation of foamy histiocytic lesion, is sometimes found during EGD, in the lamina of the gastric inflamed mucosa [7]. Gastric xanthoma is a clear indication of *H. pylori* positive and persists after *H. pylori* eradication.

GX has received little clinical significance, apparently because it is considered a benign entity. A retrospective cohort study revealed a significant association between the presence of GX and GC [5]. Also, another cohort study at the hospital itself has reported that GX was a useful marker for predicting GC development through examination of an endoscopic follow-up [4]. Some univariate analyses revealed that gastric xanthoma (GX) was considerably more common in Group B than in Group A. Moreover, multivariate logistical assessment findings suggested that GX was an ominous sign for metachronous and synchronous gastric cancer.

**Figure 1:**
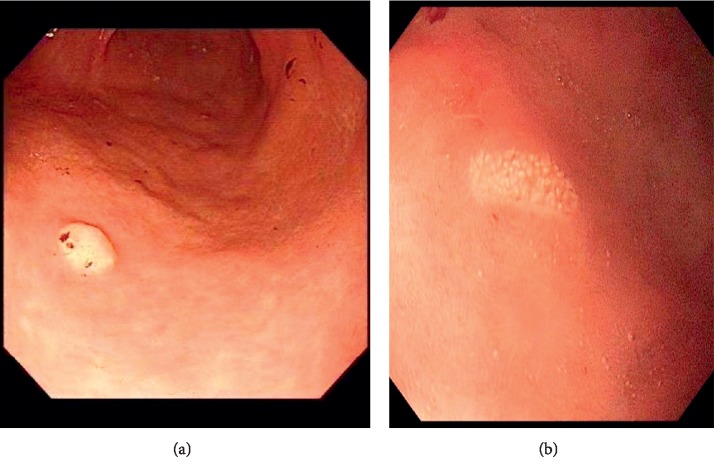
(a) Patient with gastric xanthoma in fundus, used with ME-NBI, with atrophy gastritis and erosion. (b) Another patient with gastric xanthoma and atrophy gastritis with erosion in fundus.

Nevertheless, it is remaining unclear how gastric xanthomas have participated in the development of early gastric cancer. They can be single or multiple, and women are more commonly affected [8]. Gastric xanthoma can be seen in people of all ages, but with age, the incidence of GX increases. It was reported that the mean age was 60 years [9]. Collins et al. [10], however, reported on a case of GX as young as two years old, and Halabi et al. [11] reported on a three-year-old boy with multi-GX. Gastric xanthoma found in males and females at similar rates, but some researchers reported a moderate predominance in males over females (male: female = 3.3 : 1) [12]. Also a similar study reported that males are affected more than females with a 3 : 1, by Elizabeth Bollinger (incidental funding of gastric xanthomas). Review articles written about gastric xanthomas are few. Most are case reports. In this review, we discussed the relationship between gastric xanthoma and predictive markers of gastric cancer (Figure 1).

## 2. Endoscopical Diagnosis

An upper endoscopy of the gastrointestinal tract is an examination of the upper GI tract, including the esophagus, stomach, and upper portion of the tiny intestine (duodenum). It enables a flexible tube with light and lens at the end (endoscope) to look inside these organs. This operation is sometimes referred to as an esophagogastroduodenoscopy (EGD) because all these organs are examined. If only the stomach is reviewed, it might also be called a gastroscopy. Medicine may be provided before the operation begins to make the patient sleepy not to feel any discomfort. Endoscopic diagnosing for the benign tumor-like lesions can be seen in the upper gastrointestinal examination. Gastric xanthelasmas are small size (usually <1 cm) plaques with a rough surface and yellowish-white coloration [13, 14]. Gastric xanthoma (GX) can be observed at any site in the stomach, most commonly in the antrum and pyloric regions. Gastric xanthoma (GX) is often observed in single or multiple lesions. Since GXs are asymptomatic, endoscopic assessment has been incidentally diagnosed.

Moreover, GX has a typical endoscopic appearance of yellow-white, well-marked single or multiple nodules or plaques, ranging in diameter from 1 to 10 mm [15]. Endoscopy magnification with narrow-band imaging is commonly used in the identification of gastric xanthoma because this method can have the image magnified to see it very clearly. Biopsies during upper GI endoscopy are essential for the diagnosis of gastric xanthoma and allow the rule-out of gastric tumors [16]. Human macrophage markers are favorable for the immunohistochemical assessment of bioptic samples from xanthomatous lesions, while cytokeratins are negative in the lack of gastric tumors [17].

Biopsies are tissues or cells removed from the antrum during a biopsy or where gastric xanthomas are situated in the stomach so that they can be sent to the laboratory. The laboratory report will verify whether or not the sample contains foamy cells.

## 3. Histopathological Diagnosis

Gastric xanthomas are like other xanthelasmas in the skin, consisting of large foam cells that contain a combination of lipids, including cholesterol, neutral fat, low-density lipoprotein, and low-density oxidized lipoprotein [18]. The majority of these foamy cells are histiocytes. However, when looking at the entire image, plasma cells, soft muscle cells, and Schwann cells may be engaged. For differential diagnosis, immunohistochemical studies should be used. Usually, the foamy cells in xanthomas show the marker CD68, a highly glycosylated, 110-kDa membrane protein that can be highlighted with monoclonal KP1 or PGM1 antibodies, yet has a weak cytoplasmic positivity with periodic acid–Schiff (PAS) staining. Periodic acid–Schiff (PAS) is a method of staining used to detect polysaccharides in tissues like glycogen and mucosal substances such as glycoproteins, glycolipids, and mucins. They consist, microscopically, of cohesively aggregated nests of massive periodic acid–Schiff (PAS) negative round cells with relatively small nuclei and foamy cytoplasm. The majority of cells are histiocytes, even though plasma cells, smooth muscle cells, and Schwann cells can be included in the large picture [1]. Furthermore, unique stains for cytokeratin AE1-AE3 such as Gram, Ziehl–Neelsen, Gomori methenamine silver, PAS, PAS diastase, and immunohistochemistry may be helpful [19]. They are usually 2–10 mm yellow granular or slightly elevated lesions macroscopically. Some authors suggest that gastric xanthoma may be characterized by granular or spotty configuration, as lipid islands are present between the rete ridges and are absent beneath them. The lipid islands are usually located microscopically just below the squamous epithelium [20].

## 4. Differential Diagnosis

Several GI lesions, endoscopical or histopathological, demonstrate comparable characteristics to GX. However, these lesions differ in clinical significance from GX. It is necessary to make a differential diagnosis thoughtfully.

- *Russell body gastritis (RBG)*, first mentioned in 1998 [21], is a rare inflammatory disorder characterized by abundant intramucosal polyclonal plasma cells containing immunoglobulin (Russell bodies) intracytoplasmic, eosinophilic globules that displace the nucleus, accompanied by chronic inflammatory infiltration. It generally happens in the gastric antrum. RBG endoscopic pictures show large whitish lesions and may be mistaken for xanthoma, signet ring cell carcinoma, or malignant lymphoma [22]. RBG reflects a prospective diagnostic pitfall because, for signet ring tumor cells, the distended plasma cells may be mistaken [23]. However, RBG's plasma cells lack the expression of nuclear atypia, mucicarmine, and cytokeratin. The periodic acid-ship response can assist in distinguishing Russell's bodies by providing intracytoplasmic immunoglobulins, a thick, glassy stain. The polyclonal nature of plasma cell infiltration will be demonstrated by plasma cell markers such as CD138 and CD79A and the concomitant expression of kappa and lambda light chain.
- *Signet-cell gastric adenocarcinoma* is standard xanthomas histology usually indicating periodic nuclear cells centrally situated in the foamy cells, although atypical cells can be seen in preparation for cytology. In both organizations, Masson trichrome staining may be positive. Periodic acid–Schiff staining in GX is uniformly negative and in gastric signet-cell adenocarcinoma is highly favorable [24]. Gastric xanthoma should not be confused with lipid, submucosal lipoma, pseudolipomatosis, or histiocyte accumulation without a noticeable lesion [25].
- *Pseudoxanthoma elastic (PXE)* is a progressive disease defined by the accumulation of calcium deposits in elastic fibers and other minerals (mineralization). Elastic fibers are an element of the connective tissue that gives body-wide structures power and flexibility. Mutations in the ABCC6 gene cause PXE. This gene offers directions for the production of a protein called MRP6 (also known as the protein ABCC6). This protein is mainly discovered in liver and kidney cells, with tiny quantities found in other tissues, including skin, stomach, blood vessels, and eyes. MRP6 is believed to carry certain substances across the cell membrane; however, it has not recognized the elements. Some studies indicate that the MRP6 protein stimulates the release of an unknown mechanism from cells of a molecule called adenosine triphosphate (ATP). These lesions have a high incidence of GI bleeding because of defects in the vascular component [26].
- *Xanthogranuloma* is a tumor that is macroscopically defined by the formation of numerous golden-yellow or light-yellow nodules, and the lesion is predominantly made up of foamy histiocytes mixed with chronic and acute inflammatory cells. Xanthogranuloma pathogenesis has not been fully identified, although it is believed to be a chronic lesion connected with infection, immunological illnesses, lipid transport, and lymphatic obstruction [27]. Other organs, including the gastric mucosa, colon, uterus, and pancreas, have also recorded xanthogranulomatous inflammation [28–30]. Stomach xanthogranuloma is uncommon, and to date, only a few instances have been reported [28, 31]. Oberlin first described xanthogranuloma in 1935 [27].

## 5. Risk Factors/Etiology

It has been suggested that various pathogenic mechanisms explain the presence of xanthoma cells in GX. A healing response to local trauma or inflammation is the latest concept in its etiopathogenesis. This hypothesis is defined by GX's presence, accompanied by conditions that cause stomach injury. The detailed mechanism of development, however, remains unclear. The histopathological findings for these lesions are diagnostic tools. The main diagnostic criterion is the presence of foamy histiocytes in the lamina propria differential diagnosis [13] based on immunohistochemical studies. There are several risk factors suggested for gastric xanthoma growth, including dyslipidemia, hyperplastic polyps, *H. pylori* infection, atrophy and intestinal metaplasia, gastric cancer, reflux gastritis, and immunosuppression.

### 5.1. Dyslipidemia

Dyslipidemia is one of the risk factors and is most well studied in xanthelasma growth. Cutaneous xanthomas patients have enhanced concentrations of LDL and lowered levels of HDL [32]. One research found that, in patients with high triglyceride and fasting glucose concentrations, the incidence of GX was more significant than in patients with ordinary levels [33]. In two Korean studies, dyslipidemia was discovered to represent reduced mean high-density lipoprotein (HDL) cholesterol and higher mean triglyceride concentrations compared to controls in gastric xanthelasma subjects, followed by higher body mass index (BMI) [3, 34]. Researchers reported rare instances of hyperlipidemia [35]. Two cases of severe cholestasis GX were recorded in the literature [35]. GXs vanished with cholestasis resolution in both cases (one with acute cholestasis and one with chronic cholestasis). Researchers have suggested that transient high serum lipids may cause GX formation and may vanish with cholestasis resolution. Katsu et al. noted experimental GX formation in rabbits, which was treated with chlormadinone acetate undergoing cholesterol feeding and elevated serum cholesterol concentrations. This indicates a hypercholesterolemia-GX connection. In GX patients, the greater serum lipid content may be obtained in part from circulating lipids, since excessive oxygen-free radical levels can lead to the rapid oxidation of LDL cholesterol and GX formation [13].

### 5.2. Hyperplastic Polyps

Lin et al. first reported mixed lesions showing GX and hyperplastic polyps features in 1989 [36]. Carmack et al. [36] reported that 10.3 percent of 154 instances with GX had hyperplastic polyps in research performed on the present spectrum of gastric polyps. In an unreported case, the coexistence of various GXs and hyperplastic polyps was noted. Carmack et al. [36] revealed that GX-based hyperplastic polyps were mostly <3 mm in size and discovered close to the mucosal repair site. The frequency of GX recorded as 0.3 to 3.9 percent compared to other polyps. Although GX and hyperplastic polyp etiopathogenesis and the coexistence of these two lesions are uncertain, it is proposed that they happen as an inflammatory reaction to focal mucosal damage [37].

### 5.3. Atrophy and Intestinal Metaplasia

Pathological assessment of intestinal metaplasia was performed and classified into three degrees, showing that elevated, medium, and low grade intestinal metaplasia were discovered in 28.5 percent, 17.1 percent, and 4.0 percent, respectively. GX patients experienced a significantly higher degree of endoscopic mucosal atrophic alterations than controls [13]. GX association with atrophic gastritis and intestinal metaplasia [8] has been reported in some research. The current assessment disclosed the coexistence of xanthelasma atrophy, intestinal metaplasia, and dysplasia, indicating a potential function of protumorigenesis in gastric disease. Age/sex/atrophy-matched control assessment showed in a prior prospective study that the presence of gastric xanthelasma was substantially associated with the existence of gastric cancer, suggesting that xanthelasma could serve as a warning sign of gastric cancer [5].

### 5.4. Diabetes Mellitus

One research discovered that DM was associated with gastric xanthelasma. Accumulating evidence indicates that DM plays specific roles through hyperglycemia or hyperinsulinemia in gastric carcinogenesis [37–39]. Previous studies suggest that hyperglycemia increases the output of reactive oxygen species [40, 41]. Currently, we could not explain why DM is correlated with gastric xanthelasma. However, it is tempting to speculate that excessive free radical production in hyperglycemia in patients with DM may be involved in the growth of gastric xanthelasma. In this study, it is suggested that MD is associated with gastric xanthomas; however, the reason why MD is related to gastric xanthoma is not well studied.

### 5.5. Gastric Cancer

GX is also correlated with the malignancy of gastric cancer. Muraoka et al. [42] reported on a patient with early gastric cancer with xanthoma cell proliferation, and Luk et al. [43] reported on a situation of an endoscopic and microscopic stomach carcinoid tumor that was comparable to GX. Sekikawa et al. [5] observed 50 (47.6%) cases of GX in 105 patients with gastric cancer and showed that the presence of GX in age/sex/atrophy-matched analysis was significantly associated with the presence of gastric cancer. They suggested that GX could serve as a warning sign that gastric cancer is present. Gastric damage can explain this correlation. There is an enhanced danger of gastric injury and gastric resection for both gastric cancer and gastric xanthomas. Chronic gastritis is believed to be involved in the sequence of gastric glandular atrophy and intestinal metaplasia, considered to be precursors of gastric cancer and GX. In 66 cases (72.5 percent) of all cases of gastric cancer, gastric xanthomas were present. The relationship between gastric xanthomas and the clinicopathological characteristics of gastric cancer was investigated. In reality, in advanced gastric cancer, the incidence rate of xanthomas was 61.1 percent, which was comparatively low over the same period in early gastric cancer cases (72.5 percent).

### 5.6. Reflux Gastritis

Alkaline reflux gastritis is a clinical syndrome caused by the duodenal mucosal injury. Whether bile acids, pancreatic enzymes, or alkali causes this damage is still under discussion. Alkaline reflux gastritis usually happens after surgery that most frequently interferes with the protective sphincter function of pylorus after distal gastrectomy (Billroth 1, Billroth 2) and also after simple gastrojejunostomy. The presence of GX is also correlated with bile reflux. It has been shown that the incidence of GX linked to bile reflux has increased after gastric surgery. Twenty-three years after gastric surgery, up to 60 percent of the impact of gastric xanthomas are related to the bile reflex. It has been shown that intestinal metaplasia with bile reflux increases the transport of cellular lipids [35]. It is also thought that another rare lesion, known as xanthogranulomatous gastritis (XGG), is pathogenesis caused by bile reflux. Xanthogranulomatous gastritis has histopathological results comparable to XG, but submucosal nodules in the stomach are quickly expanding [44].

### 5.7. *H. pylori* Infection

It is suggested that infection with *H. pylori* could cause a proportion of gastric xanthomas. Arima et al. observed the incidence. In patients with gastric xanthomas, *H. pylori* infection was substantially higher than that in patients without the disease [45]. Analysis of Chi-square reported a definite connection between the prevalence of xanthelasma and *H. pylori* infection rate, more atrophy, intestinal metaplasia, and dysplasia, respectively. Besides, gastric xanthelasma is a helpful marker in the development of gastric cancer in the Japanese cohort for more than three years [4]. In addition to the endoscopic diagnosis of gastric cancer, the serum CEA concentration significantly increased in the xanthelasma group. In 1996, Hori and Tsutsumi revealed *H. pylori* infection for the first time. 48% of xanthelasma biopsy specimens reported *H. pylori* infection on the surface of foveolar cells [7]. To further support that in 1999, Isomoto also recorded a close relationship between *H. pylori* infection, xanthelasma, and gastritis atrophy [13]. Similarly, this correlation was also recognized in a large cohort in Korea and it was suggested that *H. pylori* infection may cause xanthelasma [3]. The incidence of *H. pylori* infection was shown by Isomoto et al. [13]. In patients with GX, it was significantly higher than that in patients without GX (94% versus 72%).

### 5.8. Immunosuppression

The immune system is the collection of all the cells, tissues, and organs that help stave off infection from the body. Infections can become very aggressive and even fatal, without an intact immune system. Dirweesh et al. [46] revealed that xanthomas are generally asymptomatic and may remain undetected if there are no related GI lesions in the patient. Their presence may be a sign of metabolic disturbance, such as hyperlipidemia, or may be linked with other circumstances such as prior radiotherapy, chemotherapy, and infection [cytomegalovirus (CMV) and mycobacterium avium intracellulare (MAI) colitis] in immunosuppressed patients (HIV) [19]. However, the authors failed to note any evidence on how immunosuppression is correlated to gastric xanthoma. Therefore, further study is needed.

## 6. Gastric Xanthomas and Gastric Cancer Relationship

A total of 1823 patients who underwent a medical health check-up enrolled in a cohort study. In the initial endoscopic examination, 107 (5.9%) of the 1823 patients detected gastric xanthelasma. Sekikawa et al. [4] sported that gastric xanthelasma presence was significantly correlated with age more than or equal to 65 years, male gender, open-type atrophy, and diabetes mellitus presence, respectively. Also, gastric xanthelasma, characterized by lipid accumulation in histiocytic foamy cells, has been reported to be frequently observed in patients with early gastric cancer in the gastric mucosa [5]. The incidence of gastric xanthelasma in patients with gastric cancer was reported to be significantly higher [47]. Besides, in this research using endoscopic follow-up examination, it was explained that, in patients with gastric xanthelasma, the incidence of early gastric cancer was significantly higher than in those without. These findings highly indicate that gastric xanthelasma is a predictive marker in gastric cancer development. However, univariate analysis showed that not only gastric xanthelasma but also DM, gastric atrophy, and other factors were associated with the incidence of gastric cancer, and multivariate assessment confirmed that gastric xanthelasma was an independent predictive marker for the development of gastric cancer.

A retrospective cohort study recorded a significant association between the presence of GX and the existence of GC [5]. Another cohort study conducted at the same hospital reported that GX was a helpful marker for predicting GC development by conducting endoscopic follow-up examination [4]. However, both of these researches failed to explore GX as a predictive marker for metachronous and synchronous GC. Furthermore, the findings of the multivariate logistic analysis revealed that GX was a predictive marker for synchronous and metachronous GC. Shibukawa et al. [48] reported that this was the first report, to their knowledge, of the existence of GX as a helpful predictive marker for metachronous and synchronous GC. Moreover, patients with gastric xanthelasma were significantly more than those without (62.7 versus 53.1). That evidence has shown that gastric xanthoma is dramatically related to the development of gastric cancer.

## 7. Discussion

Xanthomas are rare benign lesions that appear to be associated with inflammation of the gastric mucosa. As is frequently seen in patients with early gastric cancer in the gastric mucosa, xanthelasma was thought to be a predictive biomarker of gastric adenocarcinoma pre-tumorigenesis change [4, 5]. With pathological modifications such as chronic gastritis, intestinal metaplasia, atrophic gastritis, gastric ulcer, and alterations induced by bile reflux or partial gastrectomy, lipid islands are most frequently discovered in the stomach. Phagocytosis of *H. pylori* bacteria may induce the transformation of macrophages into foamy cells that penetrate the lamina. Therefore, this recent study shows that gastric xanthoma has a relationship with gastric cancer development. This research is the first to investigate the importance of gastric xanthelasma as a marker for anticipating early gastric cancer development. On the other hand, since xanthelasma is believed to result from an inflammatory response to damage of the mucosa, or aging, it may not be unexpected that there was a strong association between the existence of gastric xanthelasma and the severity of gastric atrophy. Therefore, it was found that the presence of DM was associated with gastric xanthelasma. Accumulating evidence indicates that DM plays certain roles through hyperglycemia or hyperinsulinemia in gastric carcinogenesis [37–39]. Previous studies indicate that hyperglycemia increases the output of reactive oxygen species [40]. Currently, we could not explain why DM is correlated with gastric xanthelasma. However, it is tempting to speculate that excessive free radical production in hyperglycemia in patients with DM may be involved in the growth of gastric xanthelasma. While it remains uncertain why patients with gastric xanthelasma demonstrate a greater incidence of gastric cancer, it appears probable that both DM and gastric atrophy are at least involved in gastric carcinogenesis. Orth defined the condition first in 1887 [2]. Gastric xanthomas incidence varies from 0.23% to 7% [1, 3]. The prevalence of GXs is a variety from different studies population. Most xanthomas are rare lesions, often most common in the antral and pyloric areas. *H. pylori* infection was substantially greater than that in patients without the disease [45]. Analysis of chi-square reported a positive connection between the prevalence of xanthelasma and *H. pylori* infection rate, more atrophy, intestinal metaplasia, and dysplasia, respectively. GX association with atrophic gastritis and intestinal metaplasia [8] has been reported in some research. In 66 cases (72.5%) of all cases of gastric cancer, gastric xanthomas were present. The relationship between gastric xanthomas and the clinicopathological characteristics of gastric cancer was investigated. In reality, in advanced gastric cancer, the incidence rate of xanthomas was 61.1%, which was comparatively low over the same period in early gastric cancer cases (72.5%). The incidence of gastric xanthelasma in patients with gastric cancer was reported to be significantly higher [47]. Also, in this research using endoscopic follow-up examination, it was explained that, in patients with gastric xanthelasma, the incidence of early gastric cancer was significantly greater than that in those without. These findings highly indicate that gastric xanthelasma is a predictive marker in gastric cancer development. A retrospective cohort study recorded a significant association between the presence of GX and the existence of GC [5]. Another cohort study conducted at the same hospital reported that GX was a helpful marker for predicting GC development by conducting endoscopic follow-up examination [4]. However, both of these researches failed to explore GX as a predicative marker for metachronous and synchronous GC. Furthermore, the findings of the multivariate logistic analysis revealed that GX was a predictive marker for synchronous and metachronous GC. Shibukawa et al. [48] reported that this was the first report to their understanding of the existence of GX as a useful predicative marker for metachronous and synchronous GC. Moreover, patients with gastric xanthelasma were significantly more than those without (62.7 versus 53.1). That evidence has shown that gastric xanthoma is significantly related to the development of gastric cancer.

## 8. Conclusion

The incidence of gastric xanthelasma in patients with gastric cancer was reported to be significantly high. The findings of the multivariate logistic analysis revealed that GX was a predictive marker for synchronous and metachronous GC.
